# Circular RNA circNF1 siRNA Silencing Inhibits Glioblastoma Cell Proliferation by Promoting the Maturation of miR-340

**DOI:** 10.3389/fneur.2021.658076

**Published:** 2021-09-13

**Authors:** Li Liu, Li Jia, Jun Shao, Hanhua Liu, Qinke Wu, Xudong Wu

**Affiliations:** ^1^Taizhou First People's Hospital, Taizhou, China; ^2^Department of Urology, Yidu Central Hospital of Weifang City, Weifang, China; ^3^Jinshan Branch of Shanghai Sixth People's Hospital, Shanghai, China; ^4^Shanghai Jinshan District Central Hospital, Shanghai, China

**Keywords:** glioblastoma, circNF1, miR-340, maturation, proliferation

## Abstract

It has been reported that circNF1, a type of circular RNA (circRNA), promotes gastric cancer. This study aimed to analyze the role of circNF1 in glioblastoma (GBM). The expression of circNF1, mature miR-340, and miR-340 precursor in paired GBM and non-cancer tissues from GBM patients (*n* = 50) was analyzed by RT-qPCR. GBM cells were transfected with circNF1 siRNA, followed by the analysis of the expression of mature miR-340 and miR-340 precursor, to study the effects of circNF1 knockdown on the maturation of miR-340. The CCK-8 assay was carried out to explore the role of circNF1 and miR-340 in the proliferation of GBM cells. circNF1 expression was found to be upregulated in GBM and was correlated with patient survival. In glioma tissue, circNF1 was inversely correlated with mature miR-340, but not with the miR-340 precursor. In GBM cells, circNF1 siRNA silencing resulted in the upregulation of mature miR-340, but not the miR-340 precursor. The cell proliferation assay showed that circNF1 siRNA silencing and miR-340 overexpression decreased the proliferation of GBM cells. In addition, the miR-340a inhibitor suppressed the role of circNF1 siRNA silencing in cell proliferation. Therefore, circNF1 siRNA silencing may inhibit GBM cell proliferation by promoting the maturation of miR-340.

## Introduction

Glioblastoma (GBM), also known as grade 4 glioma, is a common intracranial malignancy with aggressive biological characteristics (1, 2). GBM is a major cause of cancer-related deaths worldwide (3). The treatment methods for GBM mainly include surgical resection, chemotherapy, and radiotherapy (4, 5). Despite these methods, the median survival time of GBM patients is lower than 18 months, and <6% of patients survive for over 5 years past diagnosis (4, 5). Aging and male sex are the major risk factors for GBM; however, the molecular pathogenesis of this cancer remains unclear, limiting the development of novel therapeutics (6).

Multiple biomarkers are involved in the pathogenesis of GBM (7, 8)—for instance, the PI3K/Akt/mTOR signaling pathway has been implicated as a potential target for GBM therapy (9–11). Recently, emerging evidence has shown that circular RNAs (circRNAs), which are characterized by single-strand RNAs with closed covalently loop structures, are critical for the development and progression of human cancers (12, 13). Although circRNAs have limited or no protein coding capacity, they can regulate gene transcription and/or translation to affect the biological functions of cancer cells (13, 14). These findings suggest that circRNAs may be promising targets for cancer therapy. In a recent study, a novel circRNA, circNF1, has been identified in gastric cancer (15). However, the expression level and the biological role of circNF1 in GBM remain unknown. As another group of endogenous non-coding RNAs, miRNAs have been reported to play a crucial role in human cancers (16, 17). A previous microarray analysis revealed the altered expression of miR-340 in GBM (18). Therefore, this study was designed and carried out to explore the interaction between circNF1 and miR-340 in GBM.

## Materials and Methods

### Tissue Acquisition

In this study, a total of 50 GBM patients (30 men and 20 women; age range, 55–70 years; median age, 63 years), who underwent surgical resection at Jinshan Branch of Shanghai Sixth People's Hospital between June 2016 and June 2018, were included. Patients were excluded if they received chemotherapy, radiotherapy, and/or other therapies before surgery. Tumor tissues and adjacent non-cancerous tissues (within 3 cm around tumors) were collected after surgery, and a histopathological examination was conducted to validate the diagnosis of GBM. This study was approved by the Ethics Committee of our institution (no. 2020032400376).

### Follow-Up

The patients received chemotherapy or radiotherapy. From the day of admission, all patients were visited every month for a total of 2 years. Follow-up data were used to perform the survival analysis. The 50 patients either completed the follow-up or died of GBM.

### GBM Cells and Transfections

U-138 and U-87 human GBM cell lines (ATCC) were used as the cell models of GBM. Fetal bovine serum (10%) was added to Eagle's minimum essential medium to cultivate both U-138 and U-87 cells at 37°C, 5% CO_2_, and 95% humidity.

circNF1 siRNA and siRNA negative control (NC), miR-340 mimic and miR-340 NC, and miR-340 inhibitor and inhibitor NC were purchased from Invitrogen (Shanghai, China). The U-138 and U-87 cells were transfected with 40 nM siRNA, miRNA, or inhibitor using Lipofectamine 2000 (Invitrogen). NC siRNA, NC miRNA, and NC inhibitor transfections were also performed as NC experiments. Untransfected cells were included in all experiments to serve as control (C) cells. Prior to the subsequent assays, the transfected cells were cultivated in fresh medium for a further 48 h.

### RNA Isolation

The U-138 and U-87 cells, as well as tissues, were used for RNA isolation, which was performed using RNAzol (Sigma-Aldrich), followed by genomic DNA removal performed by incubating with DNase I (Invitrogen) at 37°C for 80 min. Electrophoresis was performed using 5% urea-PAGE gel to analyze the RNA integrity. The RNA purity was analyzed by determining the OD 260/280 ratios.

### RT-qPCR

To analyze circNF1 expression, cDNA samples were prepared using the QuantiTect Reverse Transcription (RT) Kit (QIAGEN) with RNA samples as a template. qPCRs were carried out using SYBR Green Master Mix (Bio-Rad) with GAPDH as an internal control. To determine the expression of mature miR-340a and miR-340a precursors, RTs and qPCRs were performed using the All-in-OneTM miRNA qRT-PCR Detection Kit (GeneCopoeia). Notably, poly-(A) addition was performed prior to the analysis of mature miR-340 expression. Sequence-specific primers were used in both RTs and qPCRs to analyze the miR-340 precursor expression, and poly-(T) was used in both RTs and qPCRs to determine the expression of mature miR-340. The primer sequences used in the RT-qPCR assay are shown in Supplementary Table 1. Each experiment was performed in triplicate, and the Ct values of the target genes were normalized using the 2^−ΔΔCq^ method.

### Cell Counting Kit 8 Assay and Clone Formation Assay

A Cell Counting Kit 8 (CCK-8) assay kit (Sigma-Aldrich) was used to analyze the proliferation of U-87 and U-138 cells. Briefly, 0.1 ml medium containing 4,000 cells was added into each well of a cell culture plate (with 96 wells). At 37°C, the cells were cultivated, and CCK-8 solution was added to 10% at 2 h prior to the determination of OD (450 nm) values, which was performed every 24 h for four times. For the colony formation assay, the U-87 and U-138 cells were seeded into a six-well plate at a density of 1,000 cells/well. After 2 weeks, the GBM cells were fixed with absolute ethyl alcohol and stained with 4% crystal violet.

### Luciferase Reporter Gene Assay

Luciferase plasmids with wild or mutant circNF1 were constructed and thenco-transfected with the miR-430 mimic using Lipofectamine 2000 reagent. After 48 h, the relative luciferase activity was measured using the Dual-Luciferase Reporter Assay Kit.

### Statistical Analysis

Levels of circNF1, mature miR-340, and miR-340 precursor in paired tissues were expressed as average values of three technical PCR replicates, and data comparisons were performed by paired *t*-test. Data from cell transfection experiments were expressed as mean ± SD values of three biological replicates, and data comparisons were performed by ANOVA Tukey's test. Correlations were analyzed using the Pearson's correlation coefficient. Based on the 2-year follow-up data, survival curves were plotted, and comparisons were performed using the log-rank test. Statistical significance was set at *p* < 0.05.

## Results

### GBM Exhibited an Overexpression of circNF1 and High Levels of circNF1 Predicted Poor Survival

**Figure 1:**
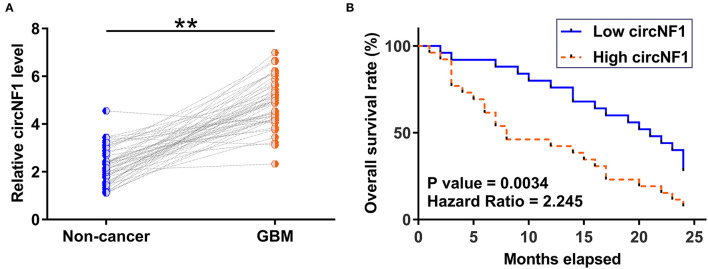
Glioblastoma (GBM) exhibited an overexpression of circNF1, and high levels of circNF1 predicted poor survival. **(A)** Paired GBM and non-cancer tissues from GBM patients (*n* = 50) were subjected to RNA isolation and RT-PCR to analyze the expression of circNF1. Levels of circNF1, mature miR-340, and miR-340 precursor in paired tissues were expressed as average values of three technical PCR replicates, and data comparisons were performed by paired *t*-test. ^**^p < 0.01. The 50 GBM patients were divided into high- and low-circNF1-level groups (*n* = 25; with a median level of circNF1 expression in GBM as a cutoff value). Based on the 2-year follow-up data, survival curves were plotted, and comparisons were performed using the log-rank test **(B)**.

Paired GBM and non-cancer tissues from GBM patients (*n* = 50) were subjected to RNA isolation and RT-PCR to analyze the expression of circNF1. The results showed that circNF1 was significantly overexpressed in GBM tissues (Figure 1A, *p* < 0.01). The 50 GBM patients were divided into high-expression (*n* = 25) and low-expression (*n* = 25) groups based on the median circNF1 expression. Table 1 shows that the clinicopathological features of the two groups were comparable. A survival analysis showed that the overall survival rate was significantly lower in the high-circNF1-level group than in the low-circNF1-level group (Figure 1B). The results of a multivariate Cox regression analysis demonstrated that the expression of circNF1 was an independent prognostic factor for GBM patients (HR: 2.888, 95% CI: 1.323-6.306, *p* < 0.01) (Table 2). The data presented here suggest that circNF1 is overexpressed in GBM and predicts poor patient survival.

**Table 1:** The correlations between circRNA NF1 expression and clinicopathological features of GBM patients.

**Factor**	**circRNA NF1**		***P*-value**
	**High expression** **(*n* = 25)**	**Low expression** **(*n* = 25)**	
**Age**			0.395
≤65	13 (52.0%)	10 (40.0%)	
>65	12 (48.0%)	15 (60.0%)	
**Gender**			0.395
Female	10 (40.0%)	13 (52.0%)	
Male	15 (60.0%)	12 (48.0%)	
**IDH1**			0.417
Wild type	20 (80.0%)	23 (92.0%)	
Mutant type	5 (20.0%)	2 (8.0%)	
**MGMT methylation**			0.069
Unmethylated	14 (56.0%)	20 (80.0%)	
Methylated	11 (44.0%)	5 (20.0%)	
**Ki-67**			0.569
<30%	10 (40.0%)	12 (48.0%)	
≥30%	15 (60.0%)	13 (52.0%)	

**Table 2:** The multivariate Cox analysis revealed the independent prognostic factors for GBM patients.

**Factor**	**Multivariate Cox analysis**	
	**HR (95%CI)**	***P*-value**
**Age (years)**		
>65 vs. ≤65	0.758 (0.346-1.658)	0.487
**Gender**		
Male vs. Female	1.224 (0.563-2.662)	0.610
**IDH1**		
Mutant vs. Wild	1.404 (0.435-4.529)	0.571
**MGMT methylation**		
Methylated vs. Unmethylated	1.108 (0.501-2.452)	0.800
**Ki-67**		
≥30% vs. <30%	2.128 (0.930-4.866)	0.074
**circRNA NF1 expression**		
High vs. Low	2.888 (1.323-6.306)	0.008

### Both Mature miR-340 and miR-340a Precursor Were Under-Expressed in GBM

**Figure 2:**
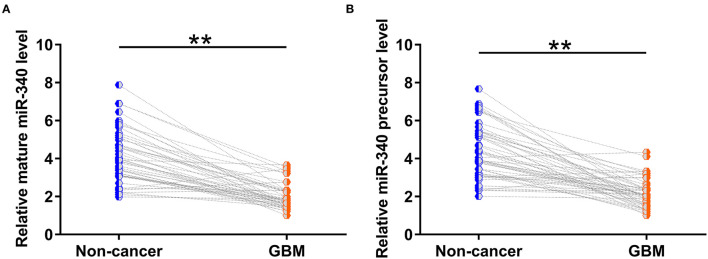
Both mature miR-340 and miR-340a precursor were under-expressed in glioblastoma (GBM). Paired GBM and non-cancer tissues from GBM patients (*n* = 50) were also subjected to RNA isolation and RT-PCR to analyze the expression of **(A)** mature miR-340 and **(B)** miR-15a precursor. Levels of circNF1, mature miR-340, and miR-340 precursor in paired tissues were expressed as average values of three technical PCR replicates, and data comparisons were performed by paired *t*-test. ^**^p < 0.05.

Paired GBM and non-cancer tissues from GBM patients (*n* = 50) were also subjected to RNA isolation and RT-PCR to analyze the expression of mature miR-340 and miR-15a precursor. The results showed that mature miR-340 (Figure 2A, *p* < 0.01) and miR-15a precursor (Figure 2B, *p* < 0.01) were significantly under-expressed in GBM tissues compared to non-cancerous tissues. Therefore, downregulation of miR-340 may be involved in GBM.

### circNF1 Was Inversely Correlated With Mature miR-340 Across GBM Tissues and circNF1 siRNA Silencing Promoted the Maturation of miR-340 in GBM Cells

**Figure 3:**
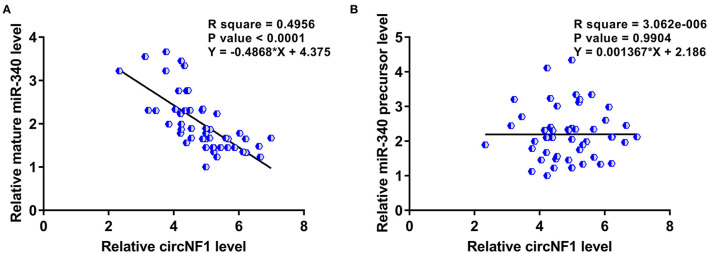
circNF1 was inversely correlated with mature miR-340 across glioblastoma (GBM) tissues. Correlations between circNF1 and **(A)** mature miR-340 or **(B)** miR-340 precursor across GBM tissues were analyzed using Pearson's correlation coefficient.

**Figure 4:**
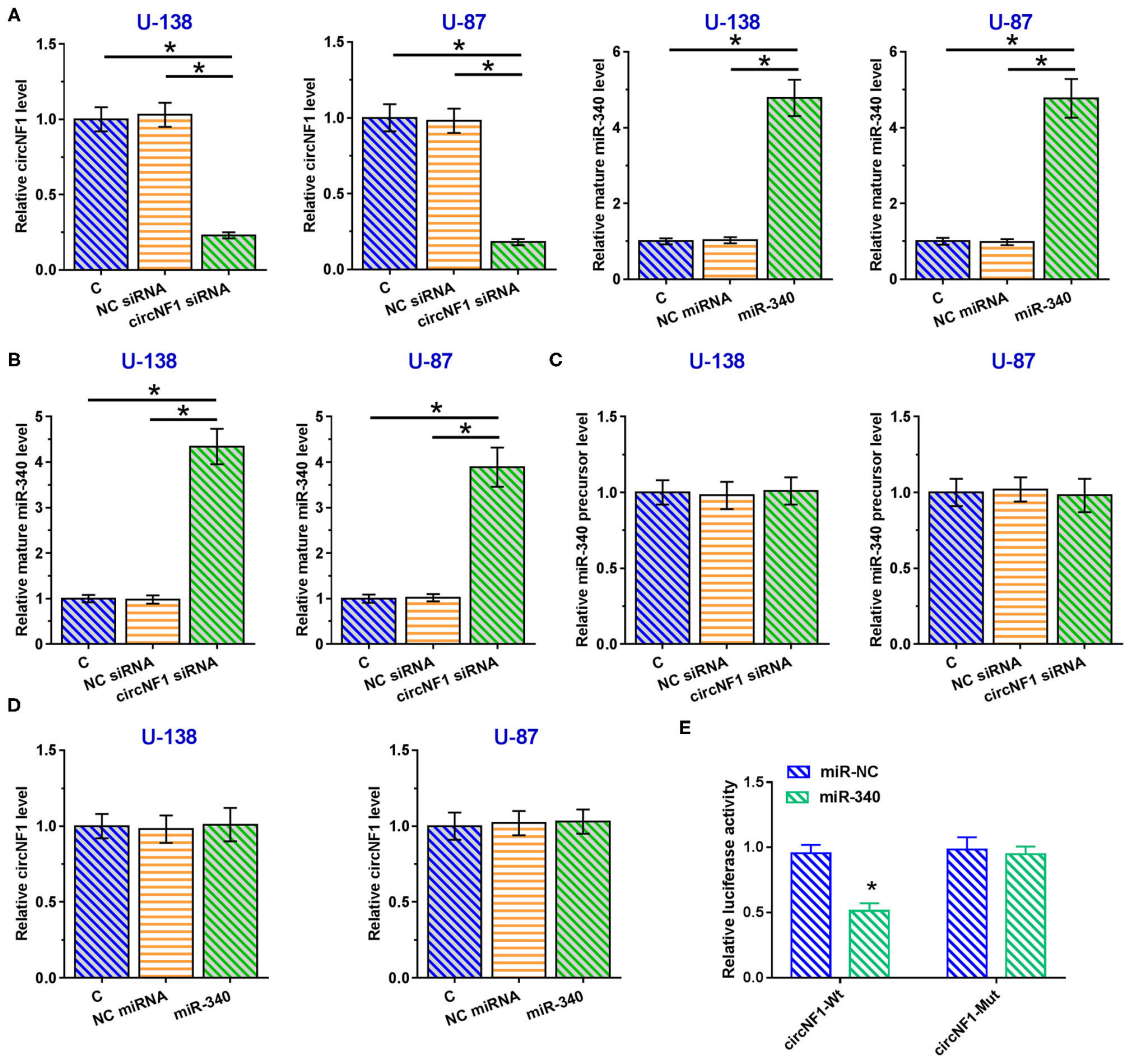
circNF1 siRNA silencing promoted the maturation of miR-340 in glioblastoma (GBM) cells. **(A)** U-138 and U-87 cells were transfected with either circNF1 siRNA or miR-340 mimic, and transfection was confirmed by RT-qPCR. The effects of circNF1 siRNA silencing on **(B)** mature miR-340 and **(C)** miR-340 precursor were explored by RT-qPCR. **(D)** The effects of miR-340 overexpression on circNF1 expression were also explored by RT-qPCR. **(E)** A luciferase reporter gene assay was conducted to determine the interaction between circNF1 and miR-340. Data from cell transfection experiments were expressed as mean ± SD values of three biological replicates, and data comparisons were performed by ANOVA Tukey's test. ^*^p < 0.05.

Correlations between circNF1 and mature miR-340 or miR-340 precursors across GBM tissues were analyzed using Pearson's correlation coefficient. The results showed that circNF1 was inversely correlated with mature miR-340 (*P* < 0.001) (Figure 3A), but not with the miR-340 precursor (*P* = 0.990) (Figure 3B) across GBM tissues, suggesting the involvement of circNF1 in the maturation of miR-340. To explore the role of circNF1 in regulating the maturation of miR-340, U-138 and U-87 cells were transfected with either circNF1 siRNA or miR-340 mimic, followed by the confirmation of transfection using RT-qPCR (Figure 4A, *p* < 0.05). In GBM cells, circNF1 siRNA silencing resulted in the upregulation of mature miR-340 (Figure 4B, *p* < 0.05), but not the miR-340 precursor (Figure 4C). Moreover, no significant changes in circNF1 expression were observed in cells overexpressing miR-340 (Figure 4D). In this study, we also conducted a luciferase reporter gene assay to determine the interaction between circNF1 and miR-340 expression. The results showed that miR-340 overexpression markedly decreased the luciferase activity of circNF1-wild but did not alter the luciferase activity of circNF1-mut (Figure 4E). These findings supported that the 3′UTR of circNF1 could be directly bound by miR-340, and circNF1 might act as a sponge for miR-340.

### circNF1 siRNA Suppressed the Proliferation of GBM Cell Through miR-340

**Figure 5:**
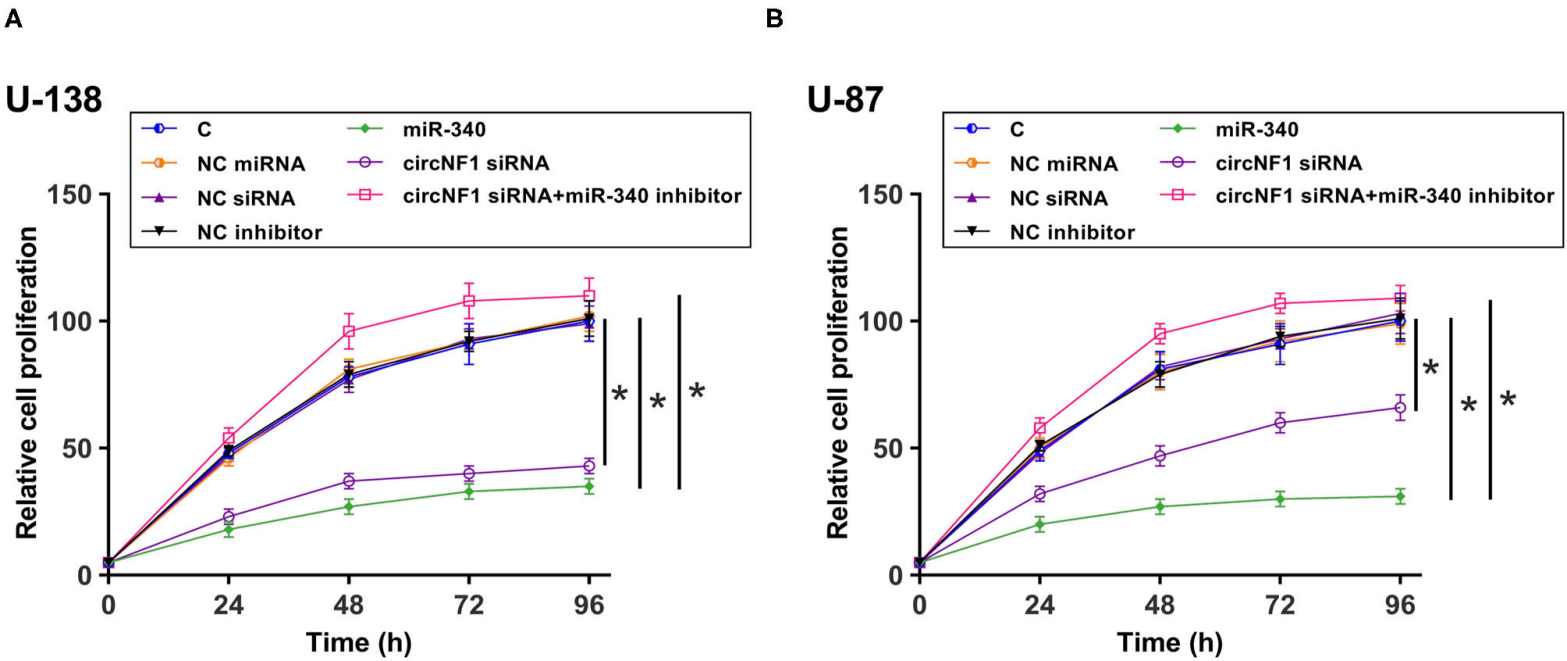
circNF1 siRNA suppressed the proliferation of GBM cell through miR-340. Cell Counting Kit 8 assay was performed to analyze the role of circNF1 and miR-340 in regulating the proliferation of U-138 **(A)** and U-87 cells **(B)**. Data from cell transfection experiments were expressed as mean ± SD values of three biological replicates, and data comparisons were performed by ANOVA Tukey's test. ^*^p < 0.05.

**Figure 6:**
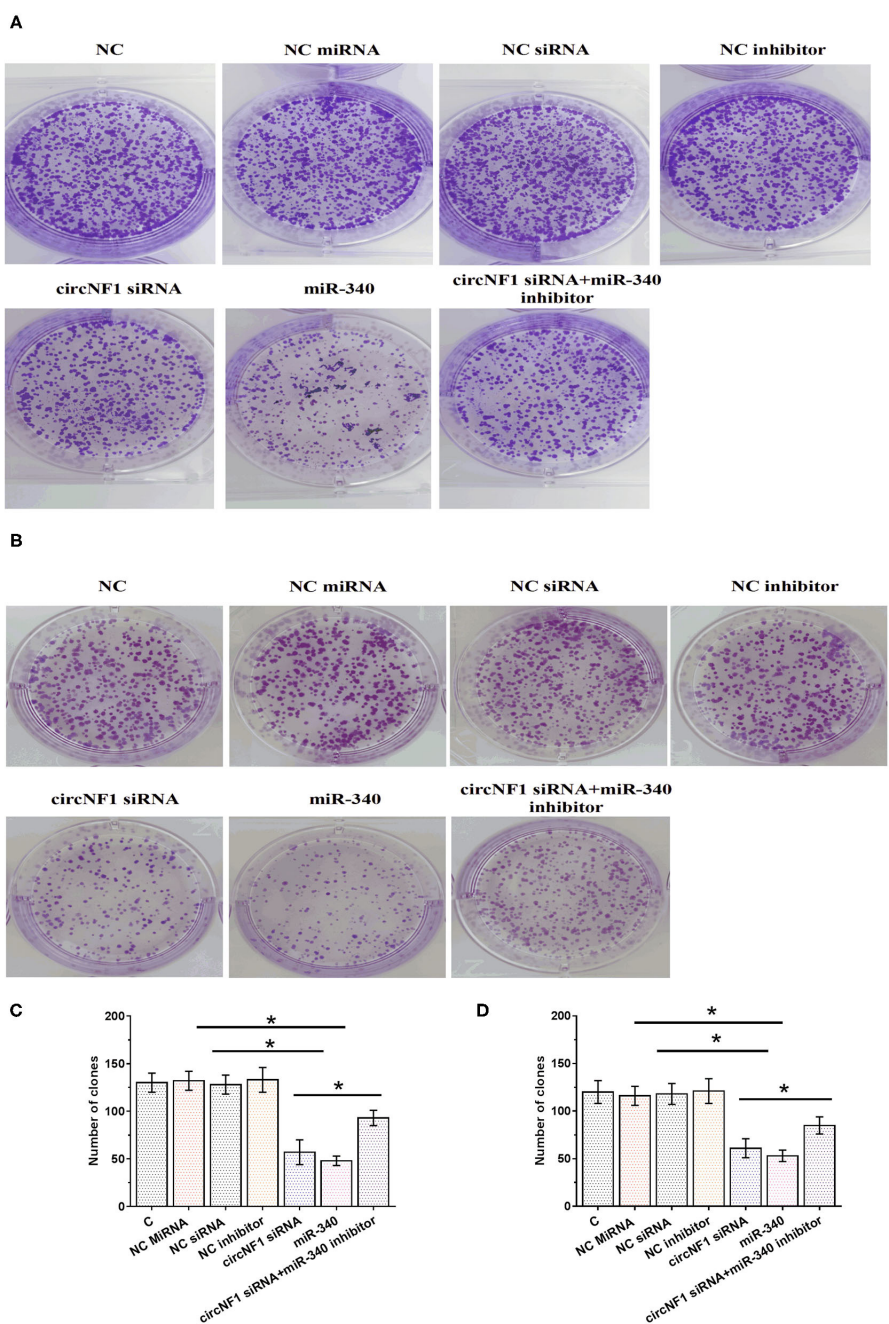
circNF1 siRNA suppressed the proliferation of glioblastoma cell through miR-340. Clone formation assay was performed to analyze the role of circNF1 and miR-340 in regulating the proliferation of U-138 **(A,C)** and U-87 cells **(B,D)**. Data from cell transfection experiments were expressed as mean ± SD values of three biological replicates, and data comparisons were performed by ANOVA Tukey's test. ^*^p < 0.05.

The CCK-8 assay was performed to analyze the role of circNF1 and miR-340 in regulating the proliferation of U-138 and U-87 cells. circNF1 siRNA silencing and miR-340 overexpression decreased the proliferation of GBM cells. In addition, the miR-340a inhibitor suppressed the role of circNF1 siRNA silencing in cell proliferation (*P* < 0.05) (Figures 5A,B). Similarly, our results showed that silencing circNF1 expression and/or miR-340 overexpression markedly reduced the number of GBM cell clones (*P* < 0.05). However, the inhibitory effects of circNF1 silencing on clone formation in GBM cells were partially reversed by the miR-340 inhibitor (*P* < 0.05) (Figure 6).

## Discussion

In this study, we explored the differential expression of circNF1 in GBM and analyzed its interactions with miR-340. Our data suggest that circNF1 is overexpressed in GBM and promotes the proliferation of GBM cells by suppressing the maturation of miR-340. In a recent study, Wang et al. reported that circNF1 expression was increased in gastric cancer samples, and circNF1 overexpression promoted the proliferation of gastric cancer cells by absorbing tumor-suppressive miR-16 (15). However, the biological roles of circNF1 in other cancers have not yet been explored. Our results demonstrated that the proliferation of GBM cells was markedly inhibited by silencing circNF1 expression. These findings suggest that circNF1 might play an oncogenic role in the development of GBM. However, animal model experiments are required to test our hypotheses.

At present, GBM patients still suffer from extremely poor survival (19). Due to the lack of classic symptoms at an early stage and of sensitive markers, the early detection of GBM is technically challenging (20). Identifying alternative biomarkers is useful for evaluating the prognosis of patients with GBM and making individualized treatment decisions. In this study, we showed that a high expression of circNF1 was significantly associated with poor survival of GBM patients, suggesting that circNF1 might be a valuable prognostic marker for GBM.

miR-340 has been reported as a tumor suppressor in many human cancers, including GBM (18), and it not only suppresses tumor growth and metastasis but also increases the sensitivity of GBM cells to chemotherapy and radiotherapy (18). In the present study, we confirmed the low expression of miR-340 in GBM and its inhibitory effect on cell proliferation. Based on our knowledge, however, the upstream regulator of miR-340 in human cancers remains unclear. Our results showed that circNF1 suppressed the maturation of miR-340 and decreased its expression level in GBM cells. These findings enrich our knowledge of the interactions between miRNAs and circRNAs. We speculated that circNF1 may affect the transportation of miR-340 precursor from the nucleus to the cytoplasm, which is required for the maturation of miR-340. However, the underlying mechanism requires further exploration.

## Conclusion

The expression of circNF1 was markedly increased in GBM, and its silencing resulted in the inhibition of cell proliferation by regulating the maturation of miR-340. These findings suggest that circNF1 may serve as a potential therapeutic target for GBM.

## Data Availability Statement

The raw data supporting the conclusions of this article will be made available by the authors, without undue reservation.

## Ethics Statement

All animal experiments were approved by the animal committee of Jinshan Branch of Shanghai Sixth People's Hospital/Shanghai Jinshan District Central Hospital. The patients/participants provided their written informed consent to participate in this study.

## Author Contributions

JS is guarantor of the integrity of the entire study. LL and LJ contributed to the study conceptualization. HL and QW contributed to the study design. XW contributed to writing and editing. All authors contributed to the article and approved the submitted version.

## Conflict of Interest

The authors declare that the research was conducted in the absence of any commercial or financial relationships that could be construed as a potential conflict of interest.

## Publisher's Note

All claims expressed in this article are solely those of the authors and do not necessarily represent those of their affiliated organizations, or those of the publisher, the editors and the reviewers. Any product that may be evaluated in this article, or claim that may be made by its manufacturer, is not guaranteed or endorsed by the publisher.
