# Supplementary material for: Circular RNA circNF1 siRNA Silencing Inhibits Glioblastoma Cell Proliferation by Promoting the Maturation of miR-340
Source: Front Neurol. 2021 Sep 13;12:658076. doi: 10.3389/fneur.2021.658076 (PMC8475906; doi:10.3389/fneur.2021.658076)
Supplement: Supplementary file 1 [file Table_1.DOCX]

**Table S1** Sequences of primers used in qRT-PCR

| Gene | Forward (5’-3’) | Reversed (5’-3’) |
| --- | --- | --- |
| circNF1 | GCAGTTTGGCCACTACAAATC | AGACATTCCTTGTTGTGCTCAG |
| miR-340 precursor | GCGGTTATAAAGCAATGAGA | GTGCGTGTCGTGGAGTCG |
| mature miR-340 | TCGGCAGGGUCAGAGUAACGA | CTCAACTGGTGTCGTGGA |
| U6 | CTCGCTTCGGCAGCACA | AACGCTTCACGAATTTGCGT |
| GAPDH | GGACCTGACCTGCCGTCTAG | GTAGCCCAGGATGCCCTTGA |
